# Research on PBFT consensus algorithm for grouping based on feature trust

**DOI:** 10.1038/s41598-022-15282-8

**Published:** 2022-07-22

**Authors:** Yong Wang, Meiling Zhong, Tong Cheng

**Affiliations:** School of Computer Science and Information Security, Guilin University of Electronic Technology, Guilin, 541004 Guangxi China

**Keywords:** Computer science, Information technology, Software

## Abstract

The consensus mechanism is the core of the blockchain system, which plays an important role in the performance and security of the blockchain system . The Practical Byzantine Fault Tolerance (PBFT) algorithm is a widely used consensus algorithm, but the PBFT algorithm also suffers from high consensus latency, low throughput and performance. In this paper, we propose a grouped PBFT consensus algorithm (GPBFT) based on feature trust. First, the algorithm evaluates the trust degree of nodes in the transaction process through the EigenTrust trust model, and uses the trust degree of nodes as the basis for electing master nodes and proxy nodes. Then, the algorithm divides the nodes in the blockchain system into multiple groups, and the consensus within each independent group does not affect the other groups, which greatly reduces the communication overhead of the consensus process when the number of nodes in the system is large. Finally, we demonstrate through theoretical and experimental analysis that the GPBFT algorithm has a significant improvement in security and performance.

## Introduction

Since Satoshi Nakamoto proposed Bitcoin^1^ in 2008, digital currencies have continued to evolve and blockchain is the underlying technology for its implementation has received much attention. The consensus mechanism is the core technology of blockchain, and in recent years, it has also received attention from academia and industry at home and abroad. The performance of blockchain systems depends on the efficiency of consensus algorithms. The existing classical consensus algorithms are proof of work (PoW) algorithm^2,3^, proof of stake (PoS) algorithm^4^, delegated proof of stack (DPoS) algorithm^5^, practical byzantine fault tolerance (PBFT) algorithm^6–8^and Raft algorithm^9,10^, etc. The core idea of PoW is to assign bookkeeping privileges and bookkeeping rewards based on the computational power of nodes. However, the PoW consensus mechanism requires a large amount of computation and therefore consumes a lot of energy with relatively low throughput. In order to solve the shortcomings of PoW consensus algorithm, some new consensus algorithms have been proposed, such as PoS consensus algorithm, DPoS consensus algorithm, PBFT consensus algorithm, etc. Although PBFT has shown good performance in terms of latency, resource requirements and node scalability, as well as node complexity, the problem of how to make the system efficiently handle a large number of nodes is a problem for PBFT, as it relies on inter-node communication, and therefore, different consensus-based PBFTs have been proposed to reduce communication costs.

Li Zhang et al.^11^ proposed a new algorithm for PBFT based on ”groups”, where all nodes are divided into groups and each group has a master node to reach a local consensus within the group based on the PBFT algorithm, and then a global consensus between groups based on the PBFT algorithm.This method can improve the communication efficiency obviously in the case of a large number of nodes, but also ignores the security performance of the algorithm.Libo Feng et al.^12^ proposed a scalable dynamic multi-intelligent hierarchical PBFT algorithm that automatically divides the layers based on the number of consensus nodes in the blockchain and the size of autonomous regions. The agent node at each layer acts as the master node, runs the PBFT algorithm and collects execution results in the autonomous region, then sends requests to the next layer and waits for the execution results. The advantage is that it significantly reduces latency, increases throughput and reduces communication costs. The disadvantage is that it increases the burden of proxy nodes. If a proxy node at one layer is not loyal, the results of other loyal nodes at the same layer will not be transmitted to the upper layer, which does not improve the security of the algorithm.Sheng Gao et al.^13^ proposed an optimised practical Byzantine fault-tolerant consensus algorithm, T-PBFT, based on the EigenTrust model, in which nodes with high trust values are constructed into A consensus group is constructed by the EigenTrust model, and the probability of group nodes being attacked is reduced by group signatures. A theoretical analysis of this method reduces communication complexity and improves consensus efficiency, but ignores the security of the algorithm.

He et al.^14^proposed an improved practical Byzantine fault-tolerant consensus algorithm , this algorithm firstly, dilutes the concept of master node and makes each node on the chain equal and efficient, and secondly, optimizes the consensus process to improve the consensus efficiency and reduces the communication overhead to half of PBFT, which increases the throughput with reduced communication overhead and latency. PBFT algorithm^15^ enables blockchains to completely disconnect from the reward mechanism of on-chain tokens and does not require a large amount of arithmetic power to maintain, and is therefore used in distributed systems, but still suffers from security vulnerabilities in the sselection of master nodes and excessive communication overhead when there are multiple nodes.At present, the optimization of PBFT consensus algorithm mainly faces the following two problems, and this paper will also optimize the PBFT consensus algorithm from the following two aspects.

- Excessive communication overhead. Blockchain is a decentralised distributed ledger database, where decentralisation means that any decision in the blockchain is voted on by all and requires a consensus process. The voting-based consensus algorithm requires a lot of communication among nodes in the case of a large consensus scale.
- Low efficiency of node consensus. Blockchain as a decentralised technology means that the distributed member nodes in the blockchain do not have a centralised and unified chief to give orders. The member nodes can only discuss among themselves to reach a consensus result, and the method and process of this discussion is determined by the consensus algorithm in the consensus layer. If there is no efficient and unified consensus mechanism among the nodes, it will lead to inefficient node consensus.

Yihao Qin et al.^16^ proposed a recursive algorithm with low computer complexity and a new method combining correlation statistical analysis and sliding window technique to detect initial faults by making full use of the information of process and quality variables.Xin Ma et al.^17^ proposed two multi-label learning algorithms for PHM of rolling bearings, named personalised binary correlation ( PBR) and hierarchical multi-label K-nearest neighbours (HML-KNN), thus converting the PHM problem into a multi-label learning problem. qing Chen et al.^18^ combined the advantages of artifificial neural network (ANN) and canonical correlation analysis (CCA) from their respective principles and proposed a novel fault detection and process monitoring method called artificial neural correlation analysis (ANCA). Based on the ideas in the above three papers, we, on the one hand, improve the efficiency and security of consensus by introducing the EigenTrust model^19^ for evaluating the trust value of nodes in the GPBFT algorithm, thus reducing the influence of malicious nodes in the consensus process. On the other hand, the consensus nodes are grouped thus reducing the communication complexity. The main contributions of this paper are as follows:

- The EigenTrust trust model is introduced to evaluate the trust value of nodes, thus reducing the influence of malicious nodes in the consensus process.
- Dividing the network-wide consensus in the original PBFT algorithm into multiple groupings for consensus, thus being able to significantly reduce the communication complexity of PBFT and improve the performance of the blockchain system.
- The view switching protocol is optimized so that when the view switching protocol is triggered, the system will elect a master node or proxy node based on the trust value size of the nodes, further enhancing the security of the system.

The rest of the paper is organised as follows. "Comparison of consensus algorithms" Section presents a comparison of consensus algorithms, "Practical Byzantine fault-tolerant algorithms" Section provides a detailed analysis of practical Byzantine fault-tolerant algorithms, and "Analysis of the advantages and disadvantages of the latest methods" Section analyses the advantages and disadvantages of existing methods for improving the PBFT algorithm; GPBFT is presented in "Methods" Section; the analysis of communication complexity and security is completed in "Results" Section; experimental testing and analysis of results are completed in "Experiments and analysis" Section; and "Conclusion" Section concludes.

## Related work

### Comparison of consensus algorithms

Consensus algorithms are highly relevant to the performance, security, and scalability of blockchain systems. PoW^20^, the first blockchain consensus algorithm used, requires nodes joining the network to compute difficulty values to compete for block-out rights, thus guaranteeing the security and fairness of bitcoin. However, PoW wastes a large amount of power in the computation process.

To address this problem, King et al.^21,22^ proposed the PoS lgorithm in PPCoin (PPC). In a PoS-based system, the age of a digital currency is equal to the product of the number of coins held and the time held, and the currency holder also receives a certain amount of income based on the age of the currency. The PoS blockchain does not rely entirely on proof of work, which effectively solves the problem of wasted PoW resources. At the same time, attackers need to hold a large amount of digital currency for a long period of time, making it much more expensive to attack. However, PoS is also more prone to forking, exposing vulnerabilities to remote attacks and disinterested attacks . How to improve the PoS algorithm has been the focus of many researchers. DPoS combines the features of PoW and PoS, where each miner can vote for a representative based on his or her interests, and the node that participates in the selection and receives the most votes wins the right to packetize the block.

Zhao et al.^23^ propose an online algorithm DPoS that is fully decentralized, low-complexity multi-resource partitioning, and also incorporates an online algorithm based on the original pairwise method and open price mechanism in the era of 5G communication, which reduces complexity, protects customer privacy, and the algorithm low overhead. Liu et al.^24^ improved the reward distribution mechanism in the PoS consensus mechanism based on the principle of calculating the Shapley value in game theory, making the reward distribution of nodes involved in generating blocks in the PoS mechanism more fair and reasonable, and also reversing the social stratification in the blockchain, thus greatly increasing the possibility of new small nodes gaining benefits.

In Algorand^25^, the VRF^26^ algorithm enables eligible users to participate in a cryptographic lottery process. The user’s account balance is proportional to the probability of being a block producer, and the nodes in the consensus group use a BFT-like^27^ algorithm to determine the final block produced. In Ourobo- ros^28^, all eligible nodes may become block producers in the next stage.Nodes publish encrypted random numbers in a given phase, then decrypt and publish random numbers in the verification phase, and finally use VRF to randomly select consensus nodes from these nodes for the next phase.

Proof of space (PoSpace)^29^, also known as proof - of-capability (PoC). It uses disk storage to prove that a user has paid for the production of a new block (new block). Specifically, the user stores a piece of data based on his public key as verifier 1. The verifier then sends multiple challenges to verifier 2 to verify that verifier 2 is storing the data honestly. Verifier 2 returns a merkle to Verifier 1 as a proof of the challenge. PoSpace is considered to be more economical and environmentally friendly than the PoW algorithm due to the efficient use of disk space.

Proof-of-Burn (PoB)^30^ is a consensus that is more closely tied to the cryptocurrency economy. It is simple and flexible and compatible with existing popular cryptocurrencies. A node burns coins in the source blockchain and then sends the coins to an unavailable address and provides the appropriate proof to be submitted to the destination blockchain for a corresponding monetary reward.

Proof of Authority (PoA)^31^ depends on permissions, in which only nodes with permissions are allowed to generate new blocks,not on the number of assets or computing power.

The Practical Byzantine Fault Tolerance algorithm, or PBFT for short^32^, which is practical solution of the BFT mechanism. Miguel Castro and Barbara Liskov^33^ proposed the PBFT algorithm in 1999 in order to improve the robustness and performance of traditional Byzantine algorithms. The PBFT algorithm is fault-tolerant to (n-1)/3, i.e. it allows no more than one-third of the nodes in the system to fail or behave badly.

We summarize the characteristics of some widely-deployed consensus mechanisms above in Table 1. In the table, we compare the transaction speed, network overhead, computing overhead, tolerated power of adversary and existing applications. From the table, we can see that the G-PBFT mechanism has advantages of high transaction speed, low network overhead, and low computing overhead over other consensus mechanisms.

**Table 1:** Comparison between consensus.

consensus	Speed	Network Overhead	Computing Overhead	Adversary Tolerance	Example of use
BFT	High	High	Low	<33.3% Replicas	Tendermint
PBFT	High	High	Low	$<3$3.3% Faulty Replicas	Hyperledger
PoB	Low	High	Low	<50% Coins	XCP
PoW	Low	High	High	<25% Computing Power	Bitcoin
PoS	Low	High	Low	<50% Stake	Peercoin
DPoS	High	Low	Low	<50% Validators	BitShares
PoA	Low	Low	Low	<50% of Online Stake	Decred
PoSpace	Low	High	Low	<50% Space	SpaceMint
GPBFT	High	Low	Low	<33.3% Endorsers	

### Practical Byzantine fault-tolerant algorithms

**Table 2:** Frequently used notation.

Notation	Definition
c	The client time identifier
o	The specific operation requested
t	The timestamp when the client initiated the request
m	The message content
d	The client message diges
v	The view number
k	The number of the current group
i	The number of the node
r	The requested action
n	The the master node assigns a number for the client request

The variable parameters commonly used in this article are shown in Table 2.

There are three roles in the PBFT algorithm: client, master and replica nodes. The client is the sender of the request and is responsible for sending the request to the master node in the format < REQUEST,o,t,c> . After the client sends the REQUEST message, the master node validates and sorts the received request message, and then broadcasts the message to the replica node. The replica nodes receive the message and reach a consensus on the consistency of the message.

For example, in the literature^33^ the Verifiable Random Function (VRF) was introduced before the third stage of PBFT consensus to further ensure the randomness of the master node selection, but as the node size increases the VRF will cause the system performance to degrade very fast. Although the above improved algorithm is clever, it still leaves Byzantine nodes in the network. The GPBFT detection-based practical Byzantine consensus algorithm proposed in this paper introduces the EigenTrust trust model to evaluate the trust value of nodes, thus reducing the influence of malicious nodes in the consensus process.

#### PBFT algorithm flow analysis:

**Figure 1:**
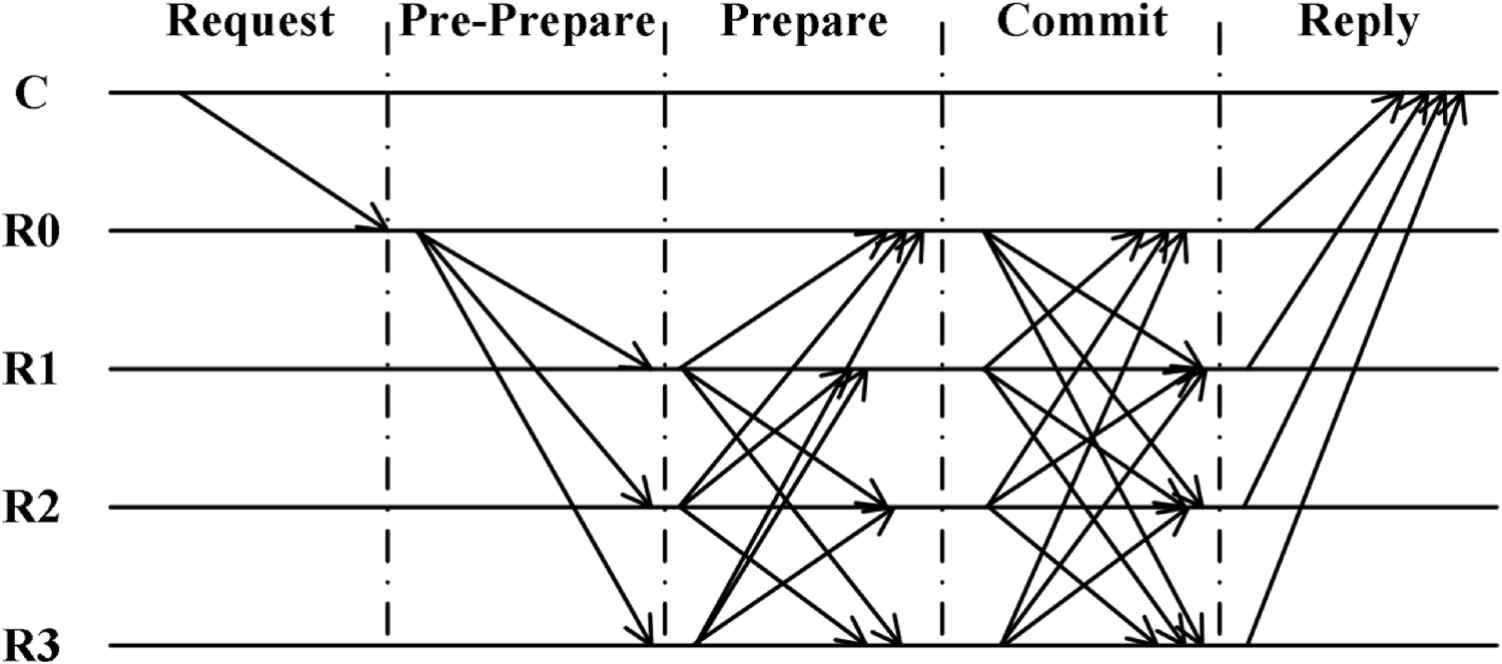
PBFT algorithm flow chart.

As shown in Fig. 1, the consensus process of the PBFT algorithm is divided into five phases, namely Request, Pre-Prepare, Prepare, Commit and Reply. The flow chart of the algorithm is shown in the diagram below.

*Request stage:* Client c sends a transaction request to master node 0.

*Pre-preparation stage:* The master node checks and processes the client’s request and then broadcasts a prep message to the replica node.

*Preparation phase:* Upon receipt of a pre-preparation message, each replica node checks its validity, adds the message to its local log, and then broadcasts a preparation message to others to indicate that it has received the suggestion and has identified it.

*Confirmation phase:* Once the node has received 2f+1 validated PREPARE messages, it sends an acknowledgment message to the other nodes.

*Response phase:* When the node enters the reply phase, the master and replica nodes reply to the client with a final feedback message.

#### PBFT algorithm view switching protocol

The PBFT algorithm triggers the view switch protocol to re-elect the master node when the master node is evil or down. The algorithm triggers the view change through a timer timeout mechanism. If the backup node does not receive a request within the specified time, the abnormal state of the master node will be detected by other nodes, at which point the replica node will override the master node through the view switch protocol. The view switching process is as follows.

The replica node detects that the master node is a Byzantine node and broadcasts a VIEW-CHANGE message to the other replica nodes with the following VIEW-CHANGE message format.

$$\begin{aligned} <VIEW -CHANGE, v +1, n, C, P, i > \end{aligned}$$

The node receives a VIEW-CHANGE message for verification validation. If the new master node receives more than 2f+1 valid messages, it is required to update the view network-wide and broadcast a NEW-VIEW message with the following message format.

$$\begin{aligned} < NEW -VIEW, v +1, V, O > \end{aligned}$$

The replica node receives the NEW-VIEW message from the master node and verifies its validity. If the verification passes, the view replacement is successful and the system will restart the unfinished consensus.

#### Inadequate PBFT algorithm

PBFT is a state machine replication algorithm. Although the algorithm solves the traditional Byzantine problem and reduces the time complexity, allowing it to be used in practical applications, the PBFT algorithm also has drawbacks and shortcomings.

The PBFT algorithm does not consider the existence of malicious nodes, but only considers the situation where the master node fails, and the master node selection method is unreasonable and does not guarantee the reliability of the master node. If the master node is malicious , it will have a great impact on the stability and security of the system.

The PBFT algorithm broadcasts messages from each node during the pre-preparation phase, the preparation phase and the confirmation phase, which consumes a very large amount of bandwidth. As the number of nodes increases, the number of communications in the consensus process also increases rapidly. Therefore the PBFT algorithm is not suitable for blockchain systems with an excessive number of nodes.

### Analysis of the advantages and disadvantages of the latest methods

Chenglin Feng et al.^34^ proposed a two-tier consensus mechanism based on PBFT. The proposed mechanism reallocates nodes into two tiers in a group, where members of the first tier act as leaders of each group in the second tier. Theoretically, this method was shown to significantly reduce the system communication complexity, but ignored the security performance and consensus efficiency of the consensus algorithm. Wenyu Li et al.^35^ proposed an optimal two-layer PBFT, i.e., a scalable PBFT-based multilayer consensus mechanism by grouping nodes into different layers and restricting intra-group communication. This method can significantly reduce the communication complexity, but ignores the security of the algorithm. YuHao Wang et al.^36^ proposed an improved consensus algorithm based on PBFT called CDBFT (Byzantine Fault-Tolerant Authorization). This algorithmic election scheme defines a voting system based on credit rewards and penalties, allowing the system cycle to be maintained over time. While this method can increase the enthusiasm of participants and reduce the enthusiasm of malicious nodes to participate in consensus, it ignores the communication complexity of the algorithm and does not improve the security of the algorithm itself significantly enough.

We, on the one hand, improve the efficiency and security of consensus by introducing the EigenTrust model for evaluating the trust value of nodes in the GPBFT algorithm, thus reducing the influence of malicious nodes in the consensus process. On the other hand, the consensus nodes are grouped thus reducing the communication complexity.

## Methods

### Holistic thinking

In response to the shortcomings of the traditional PBFT algorithm, this paper proposes a grouped PBFT consensus algorithm based on EigenTrust. The algorithm first introduces the EigenTrust trust model for evaluating the trust value of nodes, thus reducing the influence of malicious nodes in the consensus process. Secondly, the network-wide consensus in the original PBFT algorithm is divided into multiple groupings for consensus, thus being able to significantly reduce the communication complexity of PBFT and improve the performance of the blockchain system. Finally, the view-change protocol is optimised so that when the view-change protocol is triggered, the system will elect a master node or proxy node based on the trust value size of the nodes, further enhancing the security of the system.

### EigenTrust trust model

The EigenTrust model is a node trust value evaluation model that is used in the GPBFT algorithm to evaluate the trust value of each node. In the EigenTrust model, there are three ways of evaluating trust values between nodes, including direct trust values, indirect trust values and global trust values.

#### Direct trust value

The direct trust value is the trust level generated by historical interaction information between nodes. Nodes will store the history of interaction with other nodes locally and then count the number of interaction trust and the number of distrust.Each time a node communicates, the message is validated, and if it passes, the communication is satisfactory; if it fails, the communication is unsatisfactory. The number of interaction trusts from node i to node j is denoted by sat(i,j) and the number of distrusts is denoted by unsat(i,j), Sij represents the degree of trust from node i to node j and is calculated as follows.

1 $$\begin{aligned} {S_{\mathrm{{ij}}}}\mathrm{{ = sat}}\left( {i,j} \right) - unsat\left( {i,j} \right) \ \end{aligned}$$

To prevent malicious nodes from assigning higher trust levels to other malicious nodes and lower trust levels to good nodes, destabilising the system. Therefore, the local trust value needs to be normalised, and the standardised direct trust value is calculated as:

2 $$\begin{aligned} {C_{ij}} = \frac{{\max \left( {{S_{ij}},0} \right) }}{{\sum \limits _j {\max \left( {{S_{ij}}} \right) } }}\ \end{aligned}$$

#### Indirect trust value

In a blockchain system, if two nodes have not interacted directly, the trust value of one of the nodes must be calculated based on the recommendation information of the other node. For example, if node i and node j have not interacted with each other, the direct trust value cannot be calculated between the two nodes, but must be calculated by recommending node k. The trust value calculated in this way is called the indirect trust value. The formula for calculating the indirect trust value is as follows:

3 $$\begin{aligned} {C_{ij}} = \sum \limits _k {{C_{ik}}} {C_{kj}}\ \end{aligned}$$

#### Global trust values

The global trust value is the main basis for evaluating whether a node is trusted or not. Each node can calculate the global trust value from the direct trust value or the indirect trust value, which can be expressed as

4 $$\begin{aligned} T_j^{\left( {k + 1} \right) } = \sum \limits _{i - 1}^n {{C_{ij}}} T_j^{\left( k \right) }\ \end{aligned}$$

At the initialisation of the blockchain system, all nodes have the same global trust value, i.e. ${T^{\left( 0 \right) }} = 1/n$, with n denoting the number of nodes in the blockchain system. During the master node and proxy node sselection phase, nodes will be sselected based on the global trust value size.

### GPBFT algorithm flow

The GPBFT consensus algorithm divides the network-wide consensus in the original PBFT algorithm into multiple groups for consensus. There are full connections within each group in which each node can communicate with any other node by direct message passing. In whole consensus system, the proxy nodes of each group are fully connected, and the slave nodes of different groups cannot communicate with each other.

**Figure 2:**
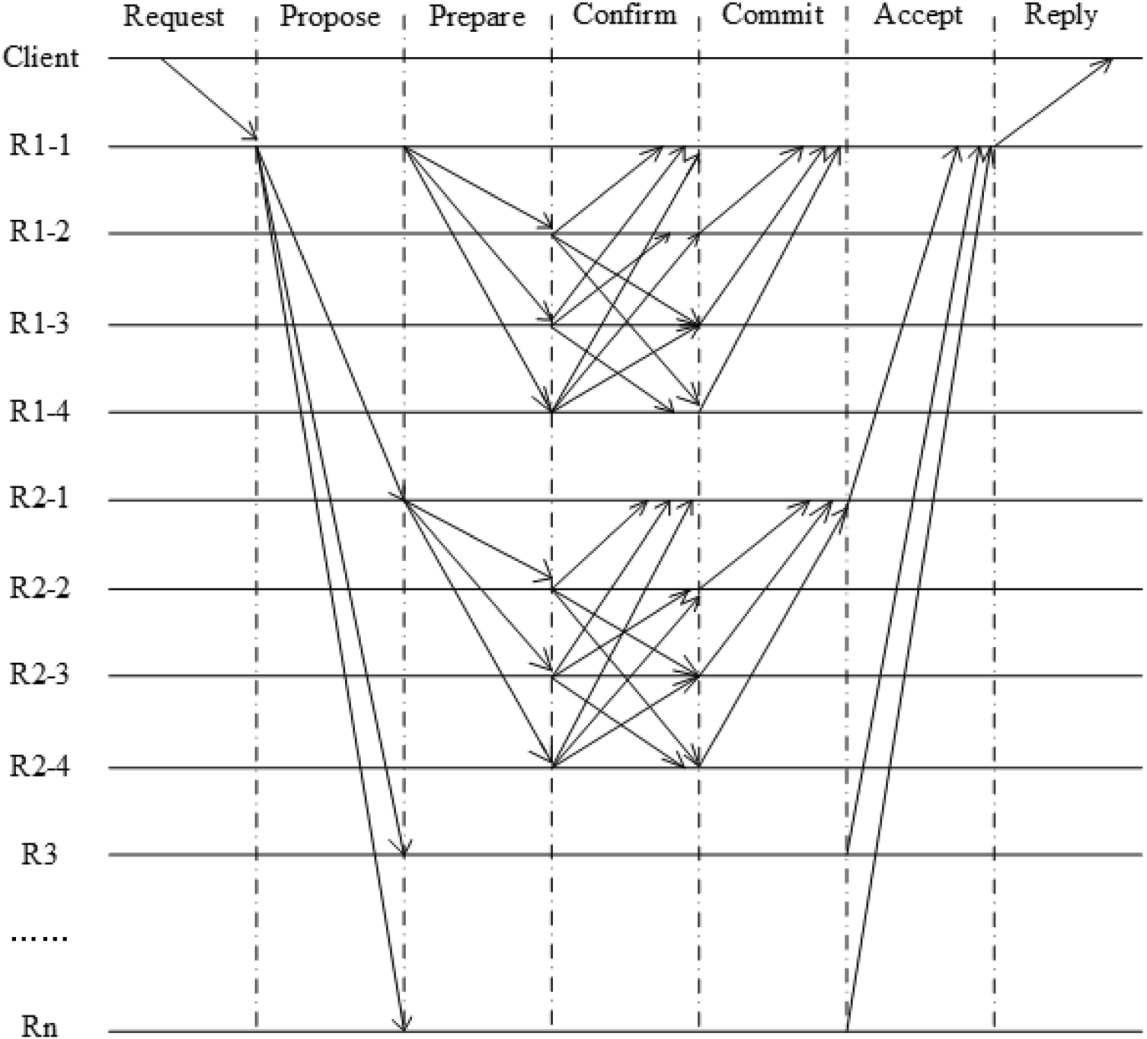
GPBFT algorithm flow chart.

As shown in Fig. 2, the GPBFT consensus algorithm process consists of seven phases: request, propose, prepare, confirm, commit, accept, and reply, which is described below.

**Figure 3:**
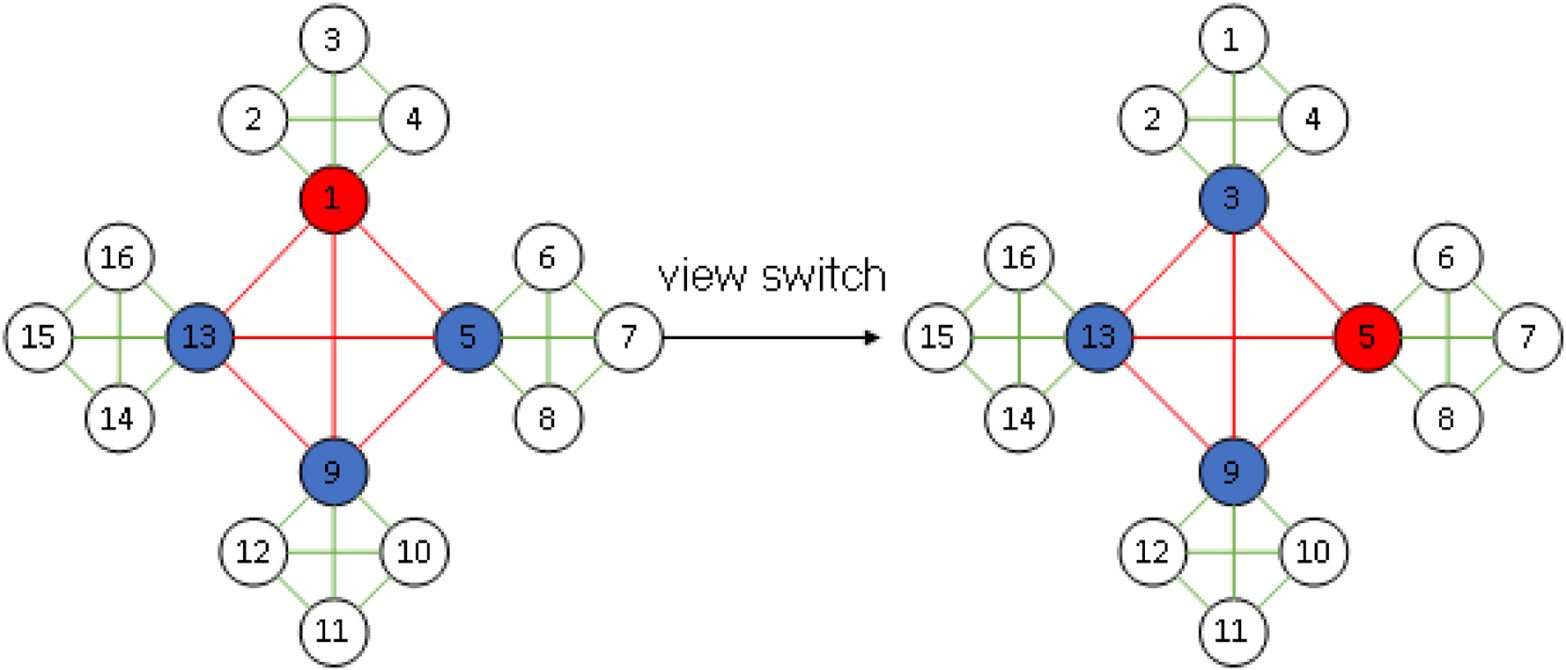
View replacement process.

Request phase: The client sends a request message < REQUEST ,o,t,c> to the master node, where REQUEST contains the message content m, and the message digest d. o indicates the specific operation requested, t indicates the timestamp when the client initiated the request, and c indicates the client time identifier.

Propose phase: After the master node receives the request from the client, it needs to verify the request, and if the verification passes, the master node assigns a number n to the client request. Then it broadcasts a $<<<$PROPOSE,v,n,d>,m> message to all proxy nodes. v is the view number, d is the client message digest, and m is the message content.

Prepare phase: The proxy node receives a PROPOSE message from the master node and checks the legitimacy of the message. If illegal, the request is discarded. If the check passes, the proxy node will send a <PREPARE ,v,n,d,k,i> message to the other other nodes in the group, where k is the number of the current group and i is the number of the node.

Confirm phase: The slave node receives the PREPARE message from the proxy node and checks the content of the message. If it agrees to the Prepare message, it then sends a <CONFIRM,v,n,d,k,i>, where i indicates the number of the node, to the other slave nodes in the group.

Commit phase: The slave node in the group receives a CONFIRM message and verifies it. If more than $2{f_k} + 1$ CONFIRM messages pass the verification, it means that more than $\frac{2}{3}$ of the nodes in the group have reached a consensus, then the slave node sends a <commit,v,n,d,k,i> to the proxy node.

Accept phase: The proxy node receives more than $2{f_k} + 1$ Commit messages will indicate that the group has reached consensus status and the proxy node will send <ACCEPT,v,n,d,k> messages to the master node.

Reply phase: The master node will send a reply request <accept, v, t, c, i, r>, where r is the requested action, to the client after receiving an accept request from more than half of the proxy nodes.

### GPBFT view switching protocol

When a replica node is corrupted or down, and the number of corrupted or down nodes does not exceed $\frac{1}{3}$ of the total number of nodes in the group, the intra-group consensus will proceed normally. Otherwise, the consensus in the group fails, which in turn causes the proxy nodes in the group to fail to participate in the consensus properly as well. If more than $\frac{1}{3}$ of the proxy nodes in the system fail to reach consensus, then the whole system will not function properly. The opposite is true.

When the master node is evil or down, the replica node will initiate a view switch request. If the view switch request was agreed, the master node will be overthrown and a new node will be re-selected as the master node. The view switch process is as follows.

a. When one of the replica nodes detects that the master node is evil or abnormal, it will broadcast a view switch message.The format of the view switch message is shown in Eq. 5, with ${V_{old}}$ representing the old view number and i representing the node number.

5 $$\begin{aligned} < VIEW - CHANGE,{V_{old}} + 1 > \end{aligned}$$

b. After receiving the message, the other replica nodes in the group verify the validity of the view switch message, and if they agree to the view switch request, they broadcast the confirmation of the replacement message to the other nodes in the group.${V_{new}}$ representing the old view number . The format of the view replacement confirmation message is shown in Eq. 6.

6 $$\begin{aligned} < VIEW - CHANGE - CONFIRM,{V_{new}},i > \end{aligned}$$

c. When the other replica nodes in the group receive $2 f _ { k } - 1$ view confirmation replacement messages, the proxy node selection is repeated.

d. When the proxy node is successfully selected, it will broadcast a view synchronization update message to other proxy nodes.

e. The other agent nodes will re-select the master node after receiving the view synchronization message and broadcast the view update message to the nodes in their respective groups. Thus, the view replacement is completed.

The Figure 3 shows the view replacement process, node 1 is the master node, when the other nodes detect the failure of node 1, the nodes in the group initiate the view replacement request, thus overthrowing the current master node, the group in which node 1 is located will re-elect the proxy node and select node 3 with the highest trust value as the proxy node. All proxy nodes then vote for the master node, and finally, node 5 with the highest trust value is selected as the master node, thus completing a view switch.

## Results

### Communication complexity analysis

In the PBFT algorithm, communication occurs in a three-phase broadcast process, which includes a pre-preparation segment, a preparation phase and an acknowledgement phase. In these three phases, nodes have to broadcast messages to other nodes in the system and also verify the messages sent from other nodes, requiring a large amount of communication between nodes in this process. In order to compare the communication complexity of the two algorithms, it is necessary to compare the number of communications between the two algorithms. In this experiment, the number of communications between the PBFT algorithm and the GPBFT algorithm are calculated and compared separately.

#### Number of PBFT algorithm communications

Assuming that there are n nodes in the system, the master node needs to broadcast a request during the prep phase, when the number of communications is n-1. In the preparation phase, each replica node has to broadcast, and the number of communications in that phase is ${\left( {n - 1} \right) ^2}$. In the acknowledgement phase, each node has to broadcast messages to the other nodes, and the number of communications in that phase is $n\left( {n - 1} \right)$. So the formula for the total number of communications for the PBFT consensus algorithm is shown in Eq. 7.

7 $$\begin{aligned} W = 2n\left( {n - 1} \right) \ \end{aligned}$$

#### Number of GPBFT algorithm communications

There are n nodes in the system, k nodes in each grouping, and the formula for calculating the number of groupings is shown in Eq. 8.

8 $$\begin{aligned} s= \frac{n}{k}\ \end{aligned}$$

The propose phase in the GPBFT algorithm is when the master node broadcasts a message to the agent node, and the number of consensus communications in this phase is $\left( {s - 1} \right)$ times. The number of communications in the intra-group consensus phase is the same as the number of communications in the PBFT algorithm, so the number of consensus communications per group in the group is $2k\left( {k - 1} \right)$, and the number of communications required by the agent node to reply to the master node in the phase is $\left( {s - 1} \right)$, so the total number of communications in the consensus process of the GPBFT algorithm is shown in Equation 9.

9 $$\begin{aligned} W = 2\left( {s - 1} \right) + 2sk\left( {\mathrm{{k - }}1} \right) \ \end{aligned}$$

#### Comparison of communication overheads

**Figure 4:**
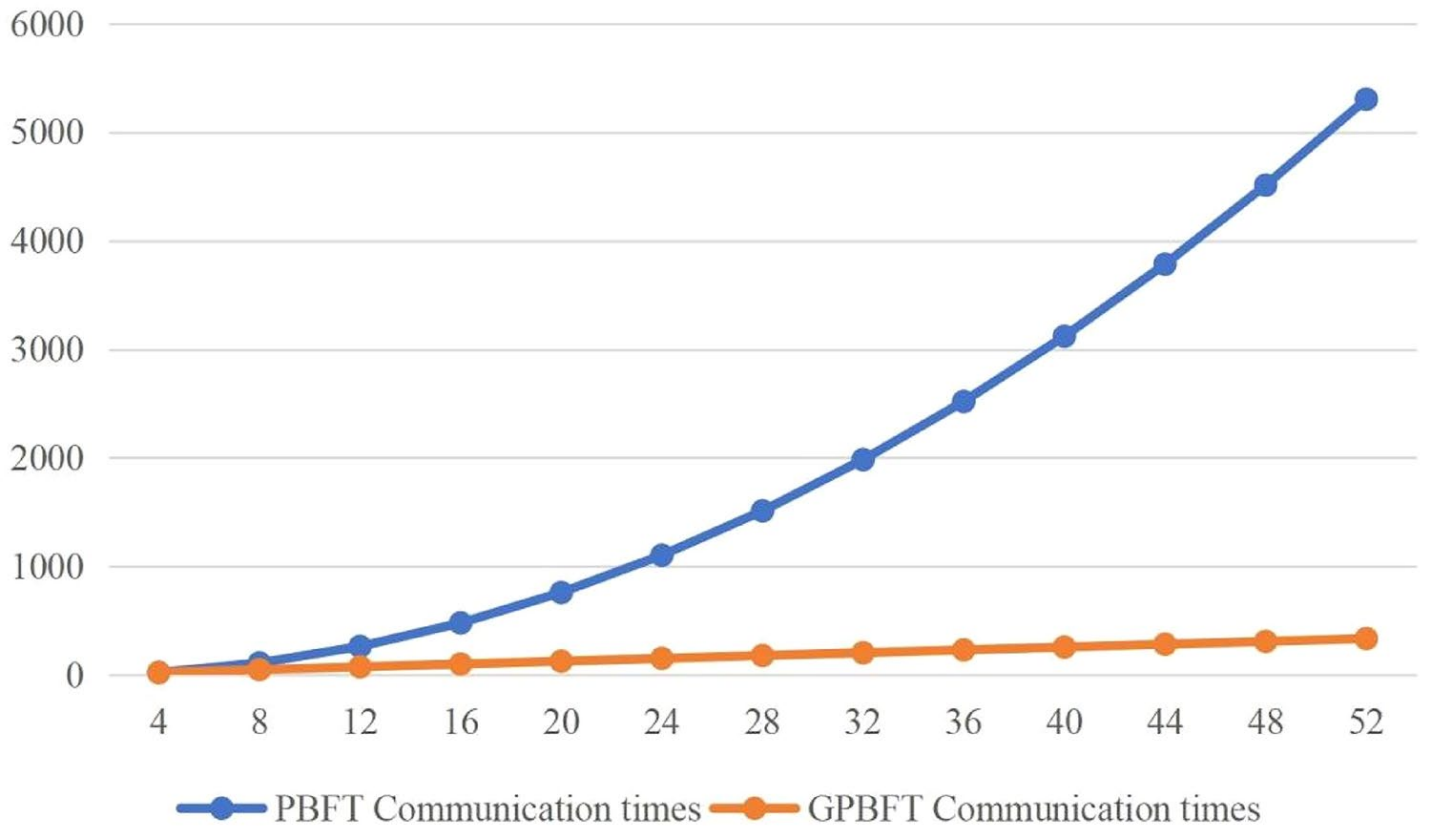
PBFT algorithm vs. GPBFT algorithm communication overhead.

**Figure 5:**
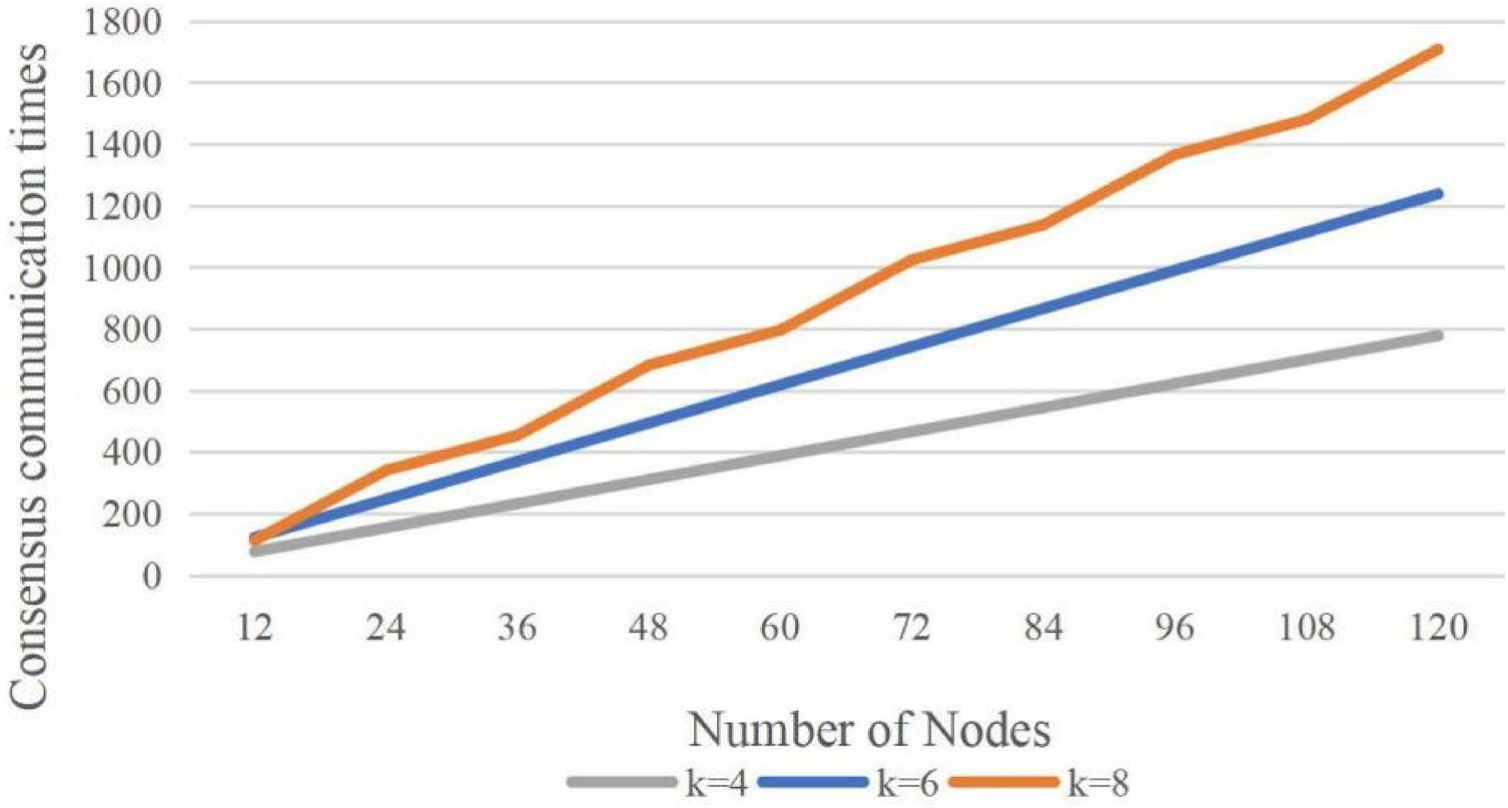
GPBFT communication overhead for different number of nodes in a group.

If the number of nodes in the group k is equal to the total number of nodes in the system n, then the number of groups $s=1$ and the number of GPBFT communications is $2k\left( {k-1} \right)$, at which point it is the case that the GPBFT algorithm and the PBFT algorithm have an equal number of communications during the consensus process. We took the case of the GPBFT algorithm with $k=4$ nodes in the group and tested the two algorithms. Figure 4 shows a comparison of the number of communications between the PBFT algorithm and the GPBFT algorithm for different numbers of nodes.

As shown in Fig. 4, the number of communications for both the PBFT algorithm and the GPBFT algorithm increases as the number of nodes increases, while the GPBFT algorithm has a significantly lower number of communications than the PBFT algorithm, that is because the number of times each node needs to broadcast increases as the number of nodes continues to increase, resulting in a rapid increase in its communications. In addition, the GPBFT algorithm uses a group consensus approach where the number of times nodes in the group need to broadcast remains the same, and as the number of nodes keeps increasing, only the number of broadcasts by the master node and the number of communications by the agent nodes in the reply phase increase. The GPBFT consensus algorithm is experimentally proven to be effective in reducing communication overhead.

In order to test the consensus communication counts of the GPBFT algorithm with different numbers of nodes in the group, the communication overheads of the GPBFT algorithm were tested at $k=4$, $k=6$ and $k=8$ respectively. The experimental comparison results are shown in Fig. 5.

A comparison of the communication overheads of the GPBFT algorithm for different numbers of nodes in a group can be seen in Fig. 5. As the total number of nodes increases, the higher the number of nodes in the group, the higher the communication overhead. This indicates that the communication overhead of the GPBFT algorithm mainly occurs in the intra-group consensus phase, so when the total number of nodes keeps increasing, it is beneficial to reduce the number of nodes in the group appropriately to improve the overall system performance.

### Security analysis

Security is an important attribute in blockchain systems, and malicious nodes are an important cause of consensus failure in consensus algorithms. Therefore, this paper introduces the EigenTrust trust model to calculate the trust value of nodes, and uses the trust value as the basis for selection. The selection based on the trust value can reduce the probability of a malicious node becoming a master or proxy node. It is inevitable that node failure or downtime will occur during the execution of the consensus algorithm, so the system must be fault-tolerant. In this section, we analyse the security of the GPBFT algorithm and discuss it in three aspects: the security of master node failure; the security of proxy node failure; and the security of replica node failure.

#### Master node failure

When the master node fails, consensus will not be completed and the master node can only be re-selected. In the event that the replica node detects abnormal behaviour in the master node, the replica node will trigger a view switch and elect a new node as a proxy node within the master node’s group according to the node’s trust value, and then the proxy node will vote to elect the master node. Once the master node selection is over, a new consensus can be initiated.

#### Agent node failure

If the number of failed agent nodes does not exceed one-third of the total number of agent nodes, then the consensus will complete normally. If the number of agent node failures continues to increase and the number of malicious agent nodes has exceeded one-third of the total number of agent nodes in the system, the consensus will not complete.

#### Copy node failure

A failure of a replica node has the least impact on the system, but the probability of a failure of a replica node is also the greatest. Both the master node and the proxy node have a higher trust value than the replica node, and a higher trust value indicates that the node is more reliable. When the number of replica node failures does not exceed one-third of the number of nodes in the group, consensus can be completed within the group. When the number of failures exceeds one-third of the total number of nodes in the group, the group will not be able to complete consensus, and only when more than one-third of the group all experience consensus failure will there be an impact on the overall system consensus.

After analysing the cases of failure of the master node, the agent node and the replica node, it is clear that the GPBFT algorithm has a greater impact on the system when the master and agent nodes fail. However, both the master node and the agent node are selected and their trust values are relatively high, so the probability of node failure is low. The GPBFT algorithm is as fault tolerant as the PBFT algorithm in the event of a failure of a replica node, and can tolerate a number of malicious nodes of $f = \left( {n - 1} \right) /3$. Therefore, the GPBFT algorithm is reliable in terms of security.

## Experiments and analysis

### Experimental environment configuration

In this paper, we use Java programming language to achieve PBFT algorithm, and on the basis of PBFT algorithm improved, GPBFT algorithm. In order to test the performance of PBFT algorithm and GPBFT algorithm, we used four PC hosts with the same configuration to conduct consensus test. The specific configuration is shown in Table 3.

**Table 3:** Experimental environment Configuration.

Environment	Configuration
CPU	Intel(R) Core(TM) i5-8500 CPU @ 3.00GHz
Memory	8G
OS	Centos 7.5
JDK	1.8

### Performance analysis

#### Time delay testing

**Figure 6:**
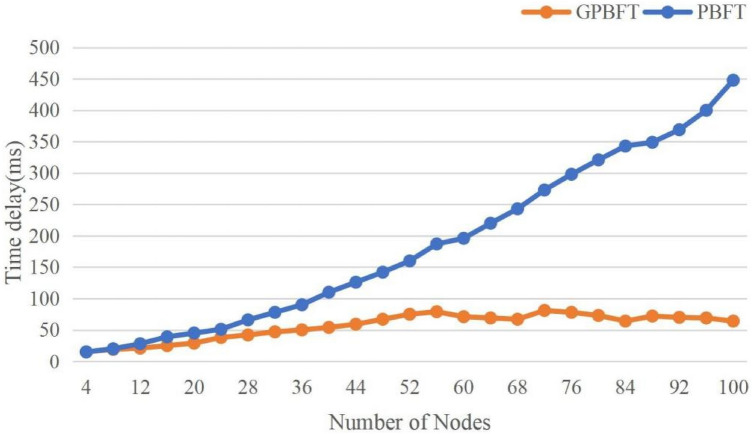
Comparison of consensus latency.

Latency refers to the time required for a consensus process, and is an important indicator of the performance of consensus algorithms. The lower the latency, the shorter the time required for consensus, and the higher the security of the blockchain. A lower latency can enable blocks to be confirmed quickly and prevents block forking. The consensus latency is calculated by the formula 10.

10 $$\begin{aligned} Delay = {T_{confirm}} - {T_{propose}}\ \end{aligned}$$

where ${T_{confirm}}$ indicates the block confirmation time and ${T_{propose}}$ indicates the start time of the proposal.The latency of PBFT algorithm and GPBFT algorithm under different nodes were tested separately under the same conditions, and 100 times of consensus were performed under each group of nodes, and then the latency obtained from 100 times of consensus was averaged. A comparison of the latency of the two algorithms is shown in Fig.  6.

**Figure 7:**
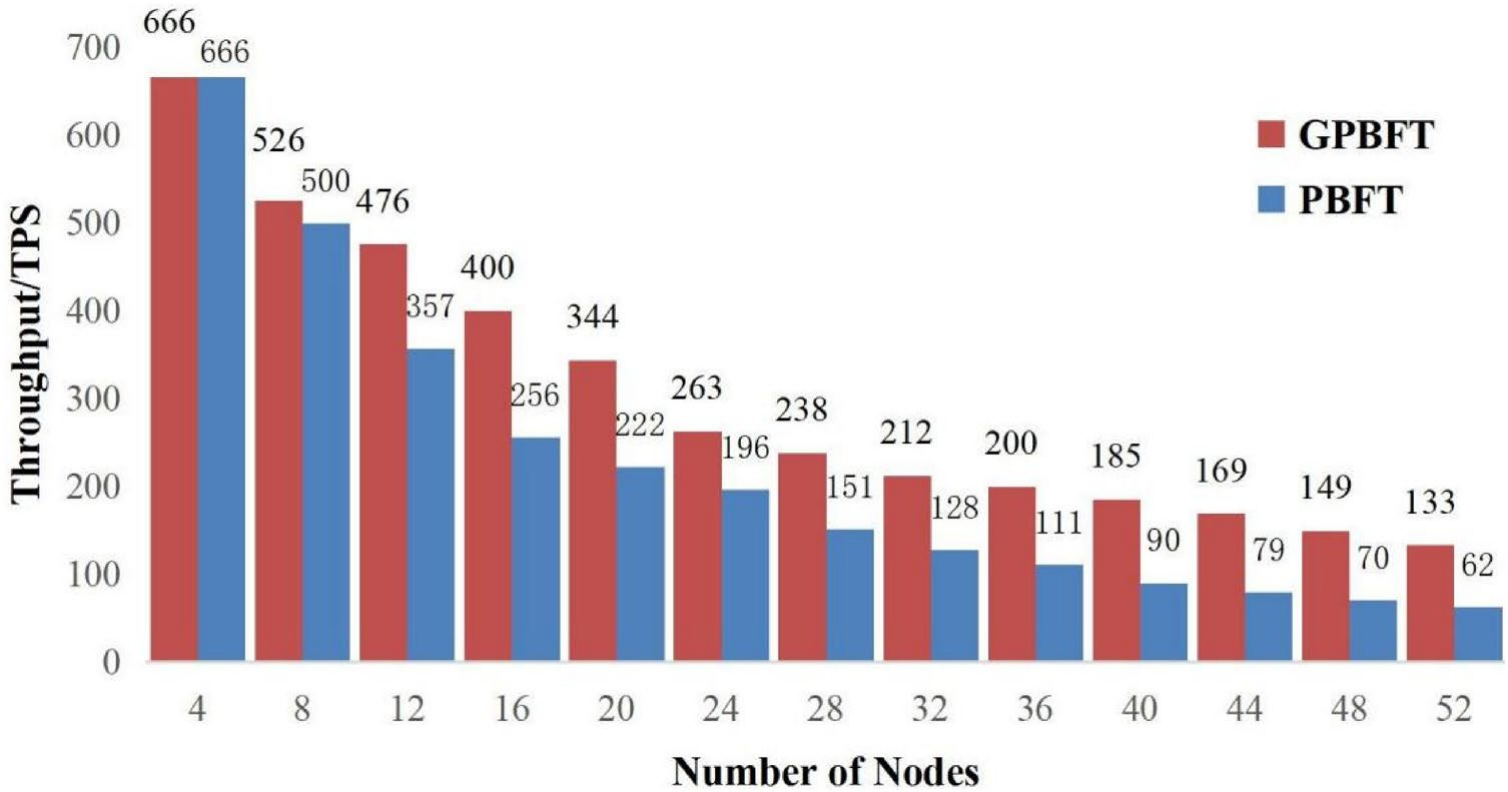
Throughput comparison.

As can be seen from the Fig. 6, the latency of both algorithms is the same when the number of nodes is 4. As the number of nodes increases, the latency of both algorithms increases, but the growth rate of the PBFT algorithm is greater than that of the GPBFT algorithm . When the number of nodes grows to a certain number, the GPBFT algorithm latency stays below 100ms and fluctuates around 90 ms. When the number of nodes reaches 100, the delay of PBFT algorithm has exceeded 400ms, while the GPBFT algorithm still remains below 100ms. Thus, it can be proved that the improved algorithm has a large improvement in latency.

#### Throughput testing

Throughput is the number of transactions completed per unit of time, and is another important indicator of consensus algorithm performance. The higher the throughput, the better the system is able to process transactions, which is generally expressed as *TPS*, i.e.

11 $$\begin{aligned} TPS = Transaction{s_{\Delta t}}/\Delta t\ \end{aligned}$$

where $\Delta t$ is the block-out time and $Transaction{s_{\Delta t}}$ is the number of transactions completed during the block-out time.The throughputs of PBFT algorithm and GPBFT algorithm were tested separately under the same conditions with different nodes. The throughput comparison of the two algorithms is shown in Fig. 7.

From the Fig. 7, it can be seen that the difference in throughput between PBFT algorithm and GPBFT algorithm is not very obvious when the number of nodes is relatively small. For example, the throughput of both PBFT and GPBFT algorithms is 666 when the number of nodes is 4. As the number of nodes increases, the throughput of both algorithms decreases, and the PBFT algorithm decreases at a significantly higher rate than the GPBFT algorithm. For example, the throughput of PBFT algorithm is 256 when the number of nodes is 16, and the throughput of GPBFT algorithm is 400. it shows that the improved algorithm has more advantages in performance in blockchain systems with more nodes.

## Conclusion

This paper first introduces the overall flow of the PBFT algorithm and the view switching protocol, and analyzes the shortcomings of the PBFT algorithm. To address the shortcomings of the PBFT algorithm, we propose a GPBFT consensus algorithm based on feature trust . Firstly, the EigenTrust trust model is introduced to evaluate the trust value of nodes, and the trust value is used as the basis for election in the election phase of master nodes as well as proxy nodes. Then, the GPBFT algorithm flow and view switching protocol are designed. Finally, the traditional PBFT algorithm and the improved algorithm GPBFT are implemented using Java language, and the two algorithms are experimentally analyzed. After the comparative analysis, the GPBFT algorithm has a large improvement in communication complexity, throughput and time delay.However, the view switching protocol needs to be carried out twice between the in-group nodes and the proxy nodes. Generally speaking, the communication cost still shows a significant decreasing trend with the increase of the number of nodes.
